# Complete chloroplast genome of *Panax japonicus* var. *major* (Burkill) C. Y. Wu & K. M. Feng (Araliaceae) and phylogenetic analysis

**DOI:** 10.1080/23802359.2019.1693917

**Published:** 2019-12-09

**Authors:** Hanya Yang, Dequan Zhang

**Affiliations:** a College of Pharmacy and Chemistry, Dali University, Dali, PR China;; b Institute of Materia Medica, Dali University, Dali, PR China

**Keywords:** Medicinal plant, *Panax japonicus* var. *major*, complete chloroplast genome, phylogenetic analysis, pharmacological activities

## Abstract

*Panax japonicus* var. *major* (Araliaceae) is an important medicinal plant, whose rhizomes have pharmacological activities such as protection of cardiovascular and cerebrovascular system, central nervous system and so on. In this study, we sequenced complete chloroplast (cp) genome sequence of *P*. *japonicus* var. *major* so as to explore phylogenetic relationship between this species and related species. The results showed that complete cp genome of *P*. *japonicus* var. *major* was 156,402 bp in length, consisting of a large single copy (LSC) region of 86,187 bp, a small single copy (SSC) region of 18,007 bp, and two inverted repeat regions (IRa and IRb) of 26,104 bp. In total, 114 genes were annotated, comprising of 80 protein-coding genes, 30 tRNA genes, and four rRNA genes. The phylogenetic analysis indicated that *P*. *japonicus* var. *major* was closely related to its original variety, *P*. *japonicus* var. *japonicus*; meanwhile, *Panax* was a monophyletic group with high support value and it had a close relationship with *Aralia* in Araliaceae.

*Panax* L., as a genus teeming with medicinal species in the world, pertains to the family Araliaceae. It is mainly distributed in East Asia and North America. The genus includes seven well-defined species in China, namely *Panax japonicus*, *Panax stipuleanatus*, *Panax zingiberensis*, *Panax notoginseng*, *Panax pseudoginseng*, *Panax ginseng*, and *Panax quinquefolius* (Xiang and Lowry 2007). Almost all the species in this genus possess important medicinal values. Their roots and rhizomes are widely used as herbal medicine in China. Ginseng Radix et Rhizoma, originating from *P*. *ginseng* is used for treatment of collapse due to Qi deficiency, fatigue, poor appetite, diarrhea, shortness of breath, diabetes, febrile diseases, insomnia, and impotence (Chen et al. 2008). It is worth noting that *P. japonicus* var. *major* also possesses similar efficacy and it is commonly used in southwest China and the Himalayan region. The roots and rhizomes of this species have been reported to contain a number of oleanane-type glycosides and exhibit a series of biological effects. For instance, it has protective cardiovascular and cerebrovascular system, central nervous system, liver, anti-inflammatory, anti-tumor, and other pharmacological activities (Zhao et al. 2010). However, until now, most of the studies for this species focused on describing its chemical compositions conclude triterpenes and triterpenoids, essential oil, steroid and steroidal saponins, flavonoids, and trace elements (Zhang et al. 2017). Herein, we reported the complete chloroplast (cp) genome sequence of *P. japonicus* var. *major* and revealed its phylogenetic relationships with related species in the Araliaceae.

Fresh leaves of *P. japonicus* var. *major* were sampled from Cangshan mountains in Dali, Yunnan, China (N25°52′10.15″, E100°01′33.04″). Meanwhile, a voucher specimen in the flowering phase was collected and deposited in the Herbarium of Medicinal Plants and Crude Drugs of the College of Pharmacy and Chemistry, Dali University (No. ZDQ17023). Genomic DNA was extracted from the dried leaves using the improved CTAB method (Doyle 1987; Yang et al. 2014), and sequenced with Illumina Hiseq 2500 (Novogene, Tianjin, China) platform with pair-end (2 × 300 bp) library. The raw data were filtered using Trimmomatic version 0.32 with default settings (Bolger et al. 2014). Then paired-end reads of clean data were assembled into circular contigs using GetOrganelle.py (Jin et al. 2018) with *P. japonicus* (No. NC_028703) as the reference. Finally, the genes were annotated using the Dual Organellar Genome Annotator (DOGMA; http://dogma.ccbb.utexas.edu/) (Wyman et al. 2004) and tRNAscan-SE (Lowe & Chan 2016). Perl script MISA (Thiel et al. 2003) was used to detect single sequence repeats (SSRs) with minimal repeat numbers of 10, 5, 4, 3, 3, and 3 for mono-, di-, tri-, tetra-, penta-, and hexa-nucleotides, respectively. The circular genome map was generated with OGDRAW version 1.3.1 (Greiner et al. 2019). The new annotated complete cp genome of this species had been submitted to NCBI database with accession number MN496312.

The complete cp genome of *P. japonicus* var. *major* was 156,402 bp in length and had a typical quadripartite structure, consisting of a large single copy (LSC) region of 86,187 bp, a small single copy (SSC) region of 18,007 bp, and two inverted repeat regions (IRa and IRb) of 26,104 bp, with 38.1% overall GC content. The cp genome contained 114 genes, including 80 protein-coding genes, 30 tRNA genes, and four rRNA genes. In the *P. japonicus* var. *major* cp genomes, 44 SSRs were detected, among which 23 (52.3%) were mono-repeats; 7 (15.9%) were di-repeats; 3 (6.8%) were tri-repeats; 8 (18.2%) were tetra-repeats and 3 (6.8%) were pentanucleotides repeats.

To investigate phylogeny of *P. japonicus* var. *major*, a total of 17 cp genome sequences from related species in Araliaceae were downloaded from the NCBI database. After using MAFFT version 7.149 for aligning (Katoh and Standley 2013), jModelTest version 2.1.7 (Darriba et al. 2012) was used to determine the best-fitting model for the dataset. Then Bayesian inference (BI) was performed by MrBayes version 3.2.6 (Ronquist et al. 2012) with two plastomes of *Diplopanax stachyanthus* (No. NC_029750; No. MG524991) as outgroups. As a result, phylogenetic analysis indicated that *P*. *japonicus* var. *major* was closely related to its original variety, *P*. *japonicus* var. *japonicus*; meanwhile, *Panax* was a monophyletic group with high support value and it had a close relationship with *Aralia* in Araliaceae (Figure 1). The complete cp genome of *P. japonicus* var. *major* would be a useful resource for studies on population genetics, molecular ecology, molecular identification, and evolution of *Panax* and related groups.

**Figure 1.**
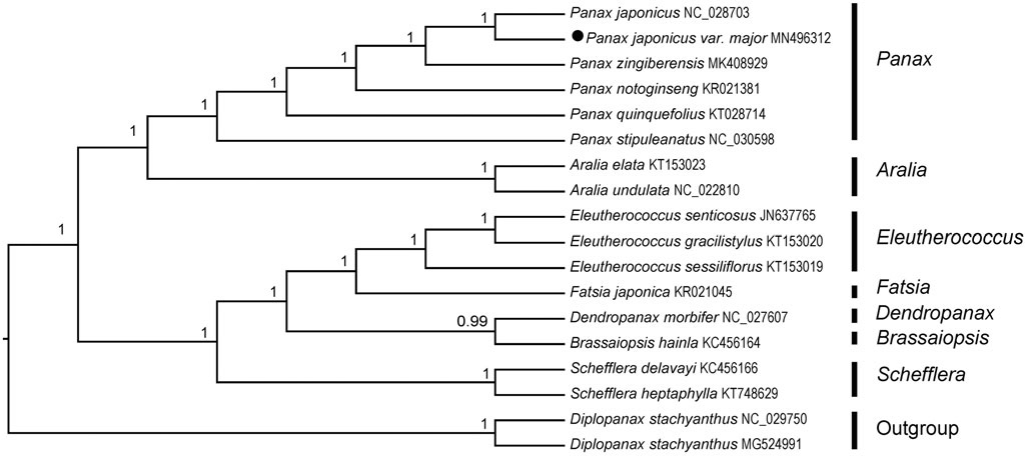
Bayesian inference (BI) tree of 18 species in the family Araliaceae based on the complete chloroplast sequences with *Diplopanax stachyanthus* as outgroup.
